# Notes and News

**Published:** 1892-01-02

**Authors:** 


### Notes and News.

Collected and Communicated.

Since the year 1883, when the Paddington Green Children's Hospital was opened, additions have been made to the out-patient department. The demands upon the hospital are now so great that it is found absolutely necessary to increase the number of cots, also to afford better accommodation for the nurses, the present arrangements being most insufficient, two of them having to sleep out. The Committee, a few years ago, purchased some land adjoining the hospital, and, after great deliberation, have decided to add a wing, for which there is ample space. The cost will probably amount to four or five thousand pounds, but they have determined not to commence building until a considerable portion of this has been raised. They trust, and in fact feel sure, this scheme will commend itself to the generous public; and they hope many will come nobly forward and assist in carrying out this excellent work, which has become imperative. 9,146 new cases were treated in the out-patient department last year, the total number of attendances being 27,624. There were also 2,491 casualities and cases of emergency. Many of these ought to have been admitted into the hospital as in-patients, but were obliged to be rejected for want of room.

Mrs. Ernest Hart writes: At this time of the year when the kind and charitable are seeking for Christmas gifts, it will, I am sure, interest many to know of a place where purchases made will benefit the worker as much as the receiver of the gift. For many years now we have been steadily at work founding and reviving industries in the midst of the poverty-stricken populations of the congested districts of Donegal, and have trained a large number of workers to produce homespuns, embroideries, wood carvings, laces, and underlinen of excellent workmanship. In the course of this training, however, much good material was spoiled for the ordinary market. We have, therefore, separated from our ordinary stock, and put on one side a large quantity of homespuns which are made of pure wool, and are thoroughly honest in manufacture, but having been made by untrained hands, are often faulty in point of pattern or design. On this account we have put them at prices far below cost, and I think if you would kindly make this fact known through the medium of your columns many benevolent persons seeking presents at this time for the poor, would be glad to know where they could purchase honest woollen stuffs at low prices, and which would wear for years. All purchases made at the Depot for Irish Industries, Donegal House, 43, Wigmore Street, London, W., help to solve the industrial question of Ireland, and to bring hope and prosperity to some Irish home.

The building scheme of the new infirmary at Derby has had to undergo some alterations. The munificent sum of £52,000 has been subscribed, and it was originally understood that an entire new building, finished and arranged according to modern requirements, could be erected at a cost of £300 per bed, and it was proposed to provide accommodation for 178 beds. This would cost £53,000. When the tenders were received for the building as desired, containing 215 beds, it was found that the estimate amounted to £113,000. To modify this large expenditure it was proposed, on the one hand, (1) to provide 104 beds in permanent wards, to erect two new and utilise two of the existing wooden huts, thus providing 178 beds, the scheme requiring an outlay of £72,000; (2) to reconstruct the existing infirmary, and provide a new out-patients' department. The advantages held out in adopting the latter scheme are increased provision of beds and accommodation for nurses, and decreased expenditure. At the special meeting of Governors on December 21st it was decided that the proposal to expend £72,000, as stated, should be adopted, and the tender of Messrs. Walker and Slater, builders, therefore, adopted. Sir E. Evans stated his intention of giving a further sum of £2,000; Sir Alfred Haslam promises £500; and Mr. Benjamin Stretton presented a cheque for £300.

The Birmingham Hospital Saturday Fund finds itself at last registered as a corporate body. Mr. Smedly had, years ago, recommended that this course should be adopted, but necessity only has brought about the result. The fund is now able to secure the £7,500 promised through the munificence of the Misses Stokes, and is entitled to establish dispensaries, convalescent homes, hospitals, and like institutions. There will be a reconstruction of the Managing Board, but this will in nowise alter the general arrangements. To quote a local paper: "The real significance of the incorporation of the collection lies in the fact that this new development is sign and signal of the anxiety of the working men to be free from the taunt levelled as they thought at their heads by the hospital abuse cry. The donations to hospitals in London from the London Hospital Saturday Collection have always been a direct payment for direct benefits. The London Committee requires full value in hospital or dispensary tickets for every sovereign subscribed. In Birmingham the Hospital Saturday donations have always been a free and an unfettered gift. And, beyond this, the artisans have specially subscribed for hospital and dispensary tickets, in order that each factory and workshop might have a right (altogether apart from Hospital Saturday claims) to any help that might be needed." One of the first acts of the fund under its new conditions is the completion of the purchase of premises for the convalescent home.

The usual Christmas entertainments are being given to the patients at the "Dreadnought" Hospital, Greenwich. The interior of the building has been decorated with evergreens, &c., and a dinner of turkeys and puddings will be served in the wards at 12.30 on Christmas Day. A concert and other amusements will also be got up for the patients during the first week in January. A social evening will also be arranged for the nurses, and a supper for the servants; besides this, there will be a Christmas tree, to which all children who have been patients during the year will be invited. There have been a larger number of patients treated in the wards of this hospital and in the out-patient department during the present year than in any year since the cholera epidemic of 1854, and there are now about 220 patients in the wards. These include representatives of the following nations: England, Sweden, Norway, Germany, Ireland, Scotland, West Indies, Portugal, Canada, Denmark, Ceylon, India, China, and Holland. There will also be a Christmas dinner given to the patients in the branch hospital at the Royal Albert and Victoria Docks, where there are sixteen patients, all of whom are suffering from severe accidents arising in the docks. Everyone in Great Britain owes much to the labour and bravery of seamen, and an earnest appeal is made for additional contributions to meet the extra expenditure caused by the much larger number of patients that have been treated in the hospitals and dispensaries of this society.

